# Discontinuation and nonpublication of clinical trials in orthopaedic oncology

**DOI:** 10.1186/s13018-024-04601-6

**Published:** 2024-02-05

**Authors:** Gurbinder Singh, Aboubacar Wague, Ayush Arora, Varun Rao, Derek Ward, Jeffrey Barry

**Affiliations:** 1 Department of Orthopaedic Surgery, University of California-San Francisco, San Francisco, CA 94143 USA; 2 Indiana University School of Medicine, Indianapolis, IN USA

**Keywords:** Orthopaedic oncology, Clinical trials, Discontinuation, Nonpublication, Intervention, Enrollment size

## Abstract

**Background:**

Despite the pivotal role of clinical trials in advancing orthopaedic oncology knowledge and treatment strategies, the persistent issues of trial discontinuation and nonpublication are significant problems. This study conducted an analysis examining clinical trial discontinuation rates, associations between intervention types and discontinuation/nonpublication, and the role of funding, enrollment size, and their implications for trial success and completion.

**Methods:**

This study, conducted on May 1, 2023, utilized a cross-sectional design to comprehensively analyze phase 3 and 4 randomized controlled trials within the realm of orthopaedic oncology. We specifically incorporated Phase 3 and 4 trials as they are designed to evaluate prolonged outcomes in human subjects and are more likely to reach publication. Study characteristics of interest included the intervention utilized in the clinical trial, presence of funding, whether the trial was published, completed, and trial enrollment size. The investigation involved an examination of ClinicalTrials.gov, a prominent online repository of clinical trial data managed by the National Library of Medicine of the USA. Descriptive statistics and multivariate logistic regressions were used to determine statistical significance.

**Results:**

Among the cohort of 130 trials, 19.2% were prematurely discontinued. Completion rates varied based on intervention type; 111 pharmaceutical trials demonstrated a completion rate of 83.8%, whereas 19 non-pharmaceutical trials exhibited a completion rate of 8.0% (*P* < .001). Surgical trials, totaling 10, showed a completion rate of 90%. The overall trial publication rate was 86.15%, with pharmaceutical interventions achieving a publication rate of 91.96%. Larger-scale trials (≥ 261 participants) emerged as a protective factor against both discontinuation (Adjusted Odds Ratio [AOR]: 0.85, 95% Confidence Interval [CI] 0.42–0.95) and nonpublication (AOR: 0.19, 95% CI 0.13–.47), compared to smaller-scale trials.

**Conclusion:**

This study accentuates the heightened vulnerability of non-pharmaceutical interventions and trials exhibiting lower rates of enrollment to the issues of discontinuation and nonpublication. Moving forward, the advancement of clinical trials necessitates a concerted effort to enhance trial methodologies, especially concerning nonpharmaceutical interventions, along with a meticulous refinement of participant enrollment criteria.

## Background

Orthopaedic oncology is a specialized branch of orthopaedic surgery that focuses on the management and diagnosis of both benign and malignant bone and soft tissue neoplasms. In the USA, the annual incidence of primary orthopaedic cancers is approximately 3970 new cases with a 5-year overall survival rate of 80% [1, 2]. Studying these conditions through clinical trials is essential for advancing knowledge, refining treatment strategies, and enhancing patient outcomes. Clinical trials have significantly progressed the field with the development of novel medical and surgical treatments, but it is still vital to understand the persistence of a concerning issue: the discontinuation and non-publication of clinical trials in orthopaedic oncology.

Discontinuation of a clinical trial entails the incompletion of a randomized controlled trial (RCT) without any identifiable justification, and this is detrimental to the progression of clinical medicine [3]. The Declaration of Helsinki has stated that the discontinuation of an RCT for any financial or personal reason may jeopardize patient safety and further contribute to unwise use of scarce scientific resources, also known as research waste [4, 5]. Trial discontinuation may also compromise the patient-physician relationship such that patients fail to access research and associated novel therapies for their complex conditions [6]. In light of these recommendations, it is noteworthy that certain randomized controlled trials (RCTs) are occasionally terminated due to preventable circumstances [6–8]. For instance, a systematic review conducted by Briel et al. revealed that a substantial 76% of all RCTs were discontinued primarily due to insufficient participant recruitment, an aspect that could potentially be addressed proactively through refined eligibility criteria, targeted outreach, and collaborative engagement strategies [9, 10]. This raises pertinent questions about the ethical considerations surrounding trial discontinuation based on practical factors such as poor enrollment. A similar investigation in Otolaryngology has shown that approximately 30% were discontinued and 40% never reached publication, with no justifications provided by the trialists [11].

Given the substantial concerns linked to clinical trial discontinuation and nonpublication, encompassing issues like research waste, potential harm to patient safety, and adverse effects on the physician–patient relationship, this study is essential for identifying preventable causes behind trial discontinuation and nonpublication [3, 5, 6, 9]. The primary objectives of this cross-sectional study are to: (1) quantitatively evaluate the rates of discontinuation and nonpublication of RCTs in the field of orthopaedic oncology; and (2) identify key factors associated with clinical trial discontinuation and nonpublication. By identifying and addressing the factors contributing to these issues, we can optimize methodological approaches to reduce research waste. We hypothesize that inadequate enrollment, financial constraints, and intervention type to be key factors influencing study discontinuation and nonpublication. This investigation holds significant clinical relevance for clinicians, researchers, and patients alike, offering valuable insights to enhance the design and completion of orthopaedic oncology research, ultimately improving patient care and outcomes.

## Methods

This investigation employed a previously utilized cross-sectional study design to assess the discontinuation and nonpublication rate of phase 3 or 4 clinical trials involving human participants in orthopaedic oncology [12–15]. We specifically incorporated Phase 3 and 4 trials as they are designed to evaluate prolonged outcomes in a cohort of human subjects, with the explicit purpose of eventual publication. Data was collected using published trial reports on ClinicalTrials.gov. These data did not involve information on human participants, so institutional review board approval was not required.

A systematic search was performed using ClinicalTrials.gov (an online repository of clinical trial data managed by the National Library of Medicine of USA) to identify eligible orthopaedic oncology clinical trials. The search was conducted on May 1, 2023, using specific search terms such as "osteosarcoma", "Ewings sarcoma", "chondrosarcoma", "Giant cell tumor of bone", "osteoblastoma", "osteochondroma", "metastatic bone cancer", and "primary bone lymphoma". The decision to use ClinicalTrials.gov was based on the directive that US clinical trialists are required to: (1) register their trial on this platform prior to study initiation; and (2) provide periodically regular updates throughout the course of the study [16]. Furthermore, each trial is assigned a unique national clinical trial (NCT) number, permitting researchers to easily identify the current status of the trial. Each clinical trial registry includes data on intervention type, recruitment status, funding, participants, and other pertinent trial information.

The present study searched for trials that were completed or discontinued for inclusion in the final analysis. All trials with the status completed were categorized to the “completed” group, and all trials with the status unknown, withdrawn, suspended, or terminated were categorized to the “discontinued” group. Trials with enrolling, not recruiting, or active status were excluded from the analysis. Studies were excluded if they were not relevant to orthopaedic oncology, were not Phase 3 or 4 trials, or if the trial was completed after May 1, 2020 due to publication lag [13, 17]. Phase 0, 1, or 2 studies were excluded, as they are not commonly intended for translation to clinical care in the context of orthopaedic oncology [16, 18, 19]. The completion date is when the final participant in the study was examined or received a treatment to collect final data for the primary aim measures, secondary aim measures, and adverse outcomes [13, 17]. No limitations were implemented for patient age or demographic information to ensure the largest sample size of randomized controlled trials. This methodological approach is in accordance with prior literature with similar analyses in numerous different scientific fields [13, 14, 20, 21].

**Fig. 1:**
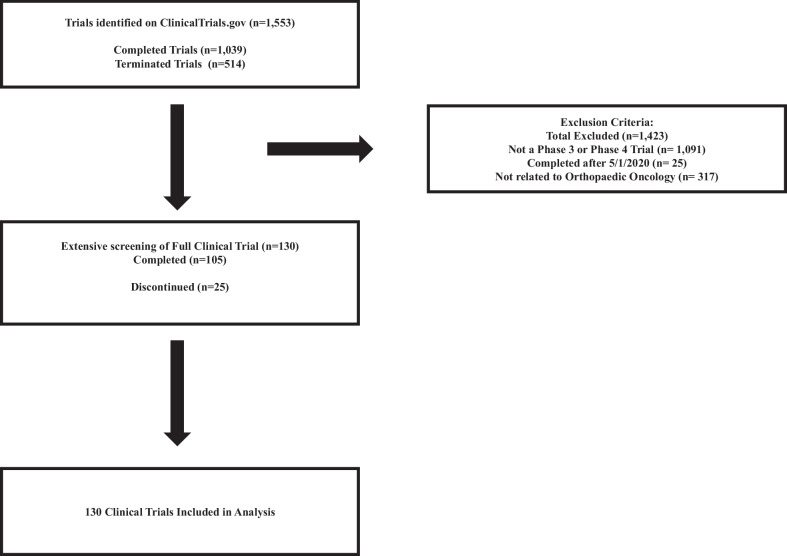
Search algorithm and exclusion criteria for identification of eligible orthopaedic oncology clinical trials

To determine the publication status of clinical trials, a team of investigators initially searched ClinicalTrials.gov. A clinical trial that linked a publication that did not report on the trial results was considered a non-published trial. A trial was considered “published” only if the associated specific trial results were available in the form of a published manuscript in a peer-reviewed journal. If no publication information was found, a search was conducted on MEDLINE via Embase, PubMed, and Google Scholar using trial authors, titles, and/or NCT numbers, as conducted in prior studies of similar nature [13–15]. This information is capitulated in Fig. 1.

Descriptive statistics, including frequencies, percentages, and the median enrollment value (interquartile range, IQR) were reported. Multivariable logistic regression was used to calculate adjusted odds ratios (AOR) to evaluate the effects of trial characteristic variables on discontinuation and publication status. Logistic regression adjusted for funding source, intervention, and enrollment size, similar to prior studies [3, 17]. Adjusted Odds Ratios (AOR) with their corresponding 95% Confidence Intervals (CI) and *p* values were calculated to determine the significance of the association. Values of AOR greater than one signified increased odds of event occurrence compared to the reference group. AOR values less than 1 indicate odds of event occurrence compared to the reference group. Statistical analysis was conducted using R programming language 4.3.1 software.

## Results

The study initially identified 1553 trials on ClinicalTrials.gov. Following an extensive screening of full clinical trials, a total of 1423 trials were excluded based on the following criteria: 1081 trials were excluded for not being Phase 3 or Phase 4 trials, 25 trials were excluded for being completed after May 1, 2020, and 317 trials were excluded for not being related to Orthopaedic Oncology. A total of 130 orthopaedic oncology clinical trials were included in the study, consisting of 105 completed trials and 25 discontinued trials (Table 1). Among the completed trials, 88 (83.8%) were pharmaceutical interventions, while 23 (92.0%) of the discontinued trials were pharmaceutical interventions. There was a significant association between intervention type and trial status (χ^2^ = 5.98, *P* < 0.001).

**Table 1:** Characteristics of completed versus discontinued trials and published versus unpublished trials (*n* = 130)

Characteristic	Total (*n* = 130)	Trial status			Publication status		
		Discontinued (25)	Completed (105)	*χ*^2^, *P*	Published (112)	Unpublished (18)	*χ*^2^, *P*
Intervention							
Pharmaceutical	111 (85.4%)	23 (92.0%)	88 (83.8%)	Pearson *χ*^2^ = 5.98, *P* < .001	103 (91.96%)	8 (44%)	Pearson *χ*^2^ = .99, *P* = .008
Behavioral/dietary	7 (5.4%)	0 (0%)	7 (5.38%)		3 (2.68%)	4 (22%)	
Device	2 (1.5%)	1 (4.0%)	1 (1.0%)		0 (0%)	2 (11.1%)	
Procedure	10 (7.7%)	1 (4.0%)	9 (8.6%)		6 (5.35%)	4 (22%)	
Funding							
NIH	50 (38.5%)	10 (40.0%)	40 (38.5%)	Pearson *χ*^2^ = .8286, *P* = .17	46 (41.1%)	4 (19.05%)	Pearson *χ*^2^ = 0.3318, *P* = .67
Industry	72 (55.4%)	13 (52.0%)	59 (56.19%)		64 (54.46%)	8 (52.38%)	
Mixed	2 (1.5%)	0 (0%)	2 (1.9%)		0 (0%)	2 (9.52%)	
Other	6 (4.6%)	2 (8.0%)	4 (3.8%)		2 (1.8%)	4 (19.05%)	
Published							
No	18 (13.8%)	4 (16%)	14 (13.3%)	Pearson *χ*^2^ = 23.64, *P* < .001	0 (0%)	18 (100%)	–
Yes	112 (86.2%)	21 (84%)	91 (86.7%)		112 (100%)	0 (0%)	
Completed							
No	25 (19.2%)	25 (100%)	0 (0%)	–	19 (16.96%)	6 (33.3%)	Pearson *χ*^2^ = 9.05, *P* < .001
Yes	105 (80.8%)	0 (0%)	105 (100%)		93 (83.03%)	12 (66.7%)	
Enrollment; median: 261 (IQR: 72–615)							
< 261	59 (45.4%)	17 (68.0%)	42 (40.0%)	Pearson *χ*^2^ = 8.72, *P* < .001	51 (45.5%)	8 (44.4%)	Pearson *χ*^2^ = 7.69, *P* < .001
≥ 261	71 (54.6%)	8 (32.0%)	63 (60.0%)		61 (54.5%)	10 (55.6%)	

This table presents the characteristics of orthopaedic oncology clinical trials, comparing completed versus discontinued trials and published versus unpublished trials. The table provides information on trial status, intervention type, funding source, and publication status, along with the corresponding statistical analyses

Regarding publication status, 112 trials were published, while 18 trials remained unpublished (Table 1). The majority of the published trials (103 out of 112, 91.96%) were pharmaceutical interventions, whereas 8 out of the 18 (44.4%) unpublished trials were discontinued. A significant association was observed between intervention type and publication status (χ^2^ = 0.99, *P* = 0.008).

Analysis of trial funding revealed that 50 trials (38.5%) were funded by the National Institutes of Health (NIH), 72 trials (55.4%) were funded by industry, 2 trials (1.5%) had mixed funding sources, and 6 trials (4.6%) had other sources of funding (Table 1). However, no significant association was found between funding source and trial discontinuation (χ^2^ = 0.8286, *P* = 0.17) or publication status (χ^2^ = 0.3318, *P* = 0.67).

For trial discontinuation, device interventions had an AOR of 1.08 (95% CI 0.78–2.35), and procedural interventions had an AOR of 1.12 (95% CI 0.63–1.26), suggesting no significant association with trial discontinuation.

Regarding funding sources, NIH-funded trials were used as the reference category. The AOR of discontinuation for industry-funded trials was 0.45 (95% CI 0.29–1.24), indicating no significant difference in trial discontinuation compared to NIH-funded trials. Trials with other funding sources had an AOR of discontinuation of 0.48 (95% CI 0.4–1.21), suggesting no significant association with trial discontinuation compared to NIH-funded trials. In terms of enrollment, trials with a recruitment below the median trial size of 261 participants were established as the reference category. The AOR of discontinuation for trials with enrollment of 261 or more participants was 0.85 (95% CI 0.42–0.95), indicating a lower likelihood of trial discontinuation compared to trials with less than 261 participants.

Similar trends were observed in the logistic regression analysis for trial nonpublication. Behavioral/dietary interventions had an AOR of discontinuation of 1.6 (95% CI 0.64–4.18), device interventions had an AOR of nonpublication of 1.03 (95% CI 0.65–4.57), and procedural interventions had an AOR of discontinuation of 0.85 (95% CI 0.57–1.93), suggesting no significant association difference with nonpublication compared to pharmaceutical trials. In the case of industry-funded trials, the AOR was 0.49 (95% CI 0.36–1.94), mixed-funded trials held an AOR of nonpublication of 0.62 (95% CI 0.27–1.87), and trials with other funding sources displayed an AOR of nonpublication of 0.74 (95% CI 0.46–2.78), signifying no significant association with nonpublication when compared to NIH-funded trials. Furthermore, trials with enrollment of 261 or more participants had an AOR of 0.19 (95% CI 0.13–0.47), indicating a significantly lower likelihood of nonpublication compared to trials with enrollment less than 261 participants.

## Discussion

In discussing the implications of our findings, it is noteworthy to highlight that no prior study has comprehensively assessed clinical trials in orthopaedic oncology with regard to discontinuation and nonpublication. Confirming our hypothesis, our cross-sectional analysis of the ClinicalTrials.gov database found a substantial rate of discontinuation among clinical trials in the field of orthopaedic oncology, with 19.2% of the evaluated trials being discontinued (Table 1). This finding was in alignment with prior discontinuation investigations in various fields of medicine, which demonstrate discontinuation rates between 10 and 30% [12, 14, 17]. High rates of discontinuation may be attributed to a variety of factors, such as recruitment difficulties, moral dilemmas, safety worries, operational challenges, and limited resources [14]. The need to appropriately discontinue a clinical trial may also arise despite the potential benefits. For instance, trials may be appropriately discontinued when novel evidence supports trial futility, when the benefit of urgent treatment is superior, and when the risks outweigh the benefits [8]. Johnson et al. revealed that about 30% of clinical trials in Otolaryngology were discontinued, and nearly half of those cases failed to offer a clear explanation for their early termination on ClinicalTrials.gov [11]. An examination of clinical trials for osteoarthritis revealed no discontinuation reasons for 97% of abandoned studies [21]. These studies underscore the prevailing trends in clinical trial discontinuation and highlight the need for a comprehensive understanding of the associated discontinuation factors to effectively address these challenges.

Our analysis revealed an association between intervention type and both trial discontinuation and nonpublication. Pharmaceutical interventions saw a higher completion and publication rate compared to other forms of interventions (Table 1). This discovery may be attributed to the extensive financial investment and industry support for clinical trials studying pharmaceutical interventions, resulting in improved completion and publishing rates [22, 23]. Conversely, interventions targeting behavior and diet demonstrated lower completion and publishing rates, pointing toward challenges unique in those areas, such as patient compliance and adherence [24–26]. Prior literature found that intrinsic motivation, cultural influences, and lifestyle factors can make it difficult for patients to adopt behaviors they can sustain over a reasonable period of time [27]. Dietary and behavioral modifications to an individual’s lifestyle and assessing the outcomes also generally require significantly longer study durations than pharmaceutical interventions [28].

No meaningful association was identified between the funding source and discontinuation or nonpublication. Despite prior literature findings suggesting an association between industry funding and trial completion and publication due to extrinsic financial rewards [23, 29], our study suggests that this factor is not the sole mediating variable. Additional elements such as experimental design, treatment administration, and organizational affiliation may have significant impact in the outcome of clinical trials [23].

The size of enrollment was a significant element contributing to the nonpublication and discontinuation. Larger-scale clinical trials were less likely to experience discontinuation and more likely to publish their results compared to smaller-scale clinical trials (Table 2). This finding concurs with prior investigations showing that larger-scale clinical trials provide more reliable and generalizable results [30, 31]. Larger trials are frequently accorded more public attention, financial resources, and require more collaborative efforts, contributing to increased odds of completion and publication [31, 32]. Because a large portion of discontinued clinical trials within our sample struggled with enrollment, we purport that clinical trialists should initially direct their attention to developing measures against this preventable reason [9, 10]. This can entail devising methodology that outlines how enrollment criteria will be met, reasonable recruitment periods, and geographic considerations for site recruitment—all factors associated with the improvement of trial completion when adequately addressed [32–36]. Axen and colleagues have further defined the challenges trialists encounter during enrollment such as the relevance of a research question to a target enrollment demographic, the importance of initially consistent and sustained communication in early stages of a trial, and the time constraints of participants [37]. With the additional consideration that primary musculoskeletal malignancies are rare, the implementation of a stepwise checklist which includes geographic variables for selection (rural vs urban), utilization of social media for recruitment outreach, and the possible need for extra incentives to increase participant motivation may help ensure adequate enrollment [37].

**Table 2:** Rates of discontinuation and non-publication of orthopaedic oncology clinical trials

Characteristic	Discontinued trials (*n* = 25)		Unpublished trials (*n* = 18)	
	No. (%)	AOR (95%CI)	No. (%)	AOR (95%CI)
Intervention				
Pharmaceutical	23 (92.0%)	1 [Ref]	8 (44%)	1 [Ref]
Behavioral/dietary	0 (0%)	–	4 (22%)	1.6 (0.64–4.18)
Device	1 (4.0%)	1.08 (0.78–2.35)	2 (11.1%)	1.03 (.65–4.57)
Procedure	1 (4.0%)	1.12 (0.63–1.26)	4 (22%)	.85 (.57–1.93)
Funding				
NIH	10 (40.0%)	1 [Ref]	4 (19.05%)	1 [Ref]
Industry	13 (52.0%)	0.45 (0.29–1.24)	8 (52.38%)	0.49 (0.36–1.94)
Mixed	0 (0%)	–	2 (9.52%)	0.62 (0.27–1.87)
Other	2 (8.0%)	0.48 (0.4–1.21)	4 (19.05%)	0.74 (0.46–2.78)
Enrollment				
< 261	17 (68.0%)	1 [Ref]	8 (44.4%)	1 [Ref]
≥ 261	8 (32.0%)	**0.85 (0.42–0.95)**	10 (55.6%)	**0.19 (0.13–0.47)**

Bold signifies stastical significance

This table examines the logistic regression for the factors associated with trial discontinuation and nonpublication in orthopaedic oncology clinical trials. The table presents the number and percentage of discontinued and unpublished trials for each characteristic, along with the Adjusted Odds Ratios (AOR) and 95% confidence intervals (CI) indicating the association between the characteristic and trial outcomes

A sufficient allocation of resources could empower investigators to overcome financial obstacles and carry out their projects, despite any operational difficulties or deficiencies in patient enrollment rates [24, 25]. Furthermore, instigating a cultural shift within the scientific sphere could be helpful to underscore the significance of publicizing both positive and negative outcomes to avoid publication bias [26]. Publication bias undermines the reliability of orthopaedic oncology research results [20, 38, 39]. Studies with favorable outcomes are more likely to be published, whereas those with mixed or unclear results are usually left unreported [20]. This paradigm warps the collective body of information, leading to a partial and potentially deceiving grasp of musculoskeletal malignancies and their treatment [13, 40]. The dissemination of only positive research results can subsequently have important implications for patient care, which could result in an overestimated appraisal of treatment success or an understatement of potential dangers and unfavorable adverse effects [40]. To foster an unbiased scientific landscape, journals could be encouraged to actively solicit submissions featuring research with uncertain or unfavorable results, thereby promoting a comprehensive understanding of the subject matter and precluding the reinforcement of biases in publication. Researchers can also be encouraged to publicize their inconclusive experimental designs and discuss outcomes from discontinued studies to avoid resource waste and promote collaboration [41–43]. This scientific approach enables us to benefit from unsuccessful attempts, prevent unnecessary trials causing resource waste, and optimize the efficiency of scientific research.

## Limitations

Although our investigation provides important knowledge regarding the discontinuation and nonpublication of orthopaedic oncology clinical tests, certain limitations persist. The relatively small number of non-pharmaceutical studies—19 in total—presents a significant constraint when attempting to conduct a robust subgroup analysis. Additionally, the rarity of musculoskeletal tumors as a disease category inherently limits the overall pool of available trials. Furthermore, the study's scope was confined to clinical trials registered on ClinicalTrials.gov, which could potentially impact the broader applicability of our findings, as our focus was primarily on trials conducted within the USA. Moreover, our reliance on publicly available information introduces the possibility of overlooking privately conducted studies or unpublished data that may be present within published reports. Lastly, the exclusion of phase 0, 1, or 2 trials inherently narrows the generalizability of our findings to the earlier phases of clinical trials, and this should be considered when interpreting our results.

## Conclusion

Our comprehensive examination of orthopaedic oncology clinical trials underlines the necessity to address the problems associated with discontinuation and nonpublication. This study highlights the elevated risks of discontinuation and nonpublication for non-pharmaceutical interventions, and trials with lower enrollment. For future studies, we recommend a concentrated effort on refining clinical trial approaches, particularly when dealing with nonpharmaceutical intervention types. There is a need to establish precise target enrollment criteria and devise a tailored systematic methodology that effectively addresses associated obstacles. By implementing these recommendations, future trials can enhance their chances of reaching completion and ensuring that their results are published to positively impact patients’ lives.

## Data Availability

The data analyzed during the present study are publicly available on ClinicalTrials.gov, and available from the first author upon reasonable request.

## Abbreviations

RCTs: Randomized controlled trials
AOR: Adjusted odds ratio
CI: Confidence interval
NCT: National clinical trial
IQR: Interquartile range
NIH: National Institutes of Health
